# Inactivation of *Wolbachia* Reveals Its Biological Roles in Whitefly Host

**DOI:** 10.1371/journal.pone.0048148

**Published:** 2012-10-29

**Authors:** Xia Xue, Shao-Jian Li, Muhammad Z. Ahmed, Paul J. De Barro, Shun-Xiang Ren, Bao-Li Qiu

**Affiliations:** 1 Department of Entomology, South China Agricultural University, Guangzhou, China; 2 Department of Genetics, University of Pretoria, Pretoria, South Africa; 3 CSIRO Ecosystem Sciences, Brisbane, Queensland, Australia; Technion-Israel Institute of Technology Haifa 32000 Israel, Israel

## Abstract

**Background:**

The whitefly *Bemisia tabaci* is cryptic species complex composed of numerous species. Individual species from the complex harbor a diversity of bacterial endosymbionts including *Wolbachia*. However, while *Wolbachia* is known to have a number of different roles, its role in *B. tabaci* is unclear. Here, the antibiotic rifampicin is used to selectively eliminate *Wolbachia* from *B. tabaci* so as to enable its roles in whitefly development and reproduction to be explored. The indirect effects of *Wolbachia* elimination on the biology of *Encarsia bimaculata*, a dominant parasitoid of *B. tabaci* in South China, were also investigated.

**Methodology/Principal Finding:**

qRT-PCR and FISH were used to show that after 48 h exposure to 1.0 mg/ml rifampicin, *Wolbachia* was completely inactivated from *B. tabaci* Mediterranean (MED) without any significant impact on either the primary symbiont, *Portiera aleyrodidarum* or any of the other secondary endosymbionts present. For *B. tabaci* MED, *Wolbachia* was shown to be associated with decreased juvenile development time, increased likelihood that nymphs completed development, increased adult life span and increased percentage of female progeny. Inactivation was associated with a significant decrease in the body size of the 4^th^ instar which leads us to speculate as to whether *Wolbachia* may have a nutrient supplementation role. The reduction in nymph body size has consequences for its parasitoid, *E. bimaculata*. The elimination of *Wolbachia* lead to a marked increase in the proportion of parasitoid eggs that completed their development, but the reduced size of the whitefly host was also associated with a significant reduction in the size of the emerging parasitoid adult and this was in turn associated with a marked reduction in adult parasitoid longevity.

**Conclusions/Significance:**

*Wolbachia* increases the fitness of the whitefly host and provides some protection against parasitization. These observations add to our understanding of the roles played by bacterial endosymbionts.

## Introduction

Endosymbiotic, mutualistic and parasitic bacterial endosymbionts that have either obligate or facultative relationships with insects, play an important role in many aspects of insect biology and ecology [1]. Knowledge drawn from the study of these associations is already leading to novel means by which to control insects and the pathogens they transmit [2], [3]. A large number of herbivorous insects, especially the phloem feeding insects of the Hemiptera suborder Sternorrhyncha (aphids, whiteflies, psyllids, scales and mealybugs) harbour these bacteria [4], which are defined as being either primary or secondary endosymbionts. Primary endosymbionts are confined to specialized host cells called bacteriocytes that together form an organ called a bacteriome [5]. They have an obligatory relationship with the host, are transmitted vertically from mother to offspring and are thought to synthesize essential non-dietary metabolites [1], [6]–[8]. Secondary endosymbionts can also occur in the bacteriocytes, but are often also found in the cells of tissues throughout the host [5], [9]. These bacteria usually have a facultative relationship with the host and while primarily transmitted vertically from mother to offspring, may also be transmitted horizontally through direct and indirect contact with other infected individuals [1], [2], [9], [10]. In many cases their function is unclear, but they are perhaps best known for the ability to act as reproductive manipulators [2], [11].

Among the secondary endosymbionts, *Wolbachia* is perhaps the most widely distributed within arthropods and has been estimated to infect as many as 70% of all species [12], [13]. *Wolbachia* can be either mutualistic or parasitic. Mutualistic functions vary and examples include the provision of essential nutrients to bedbug hosts [14], increasing fitness in a leaf-mining moth [15], uzi fly [16] and mosquito [17] although these effects are at times linked to environmental conditions [18]. *Wolbachia* can also suppress the replication of a range of RNA viruses in *Drosophila, Culex quinquefasciatus* and *Aedes aegypti* [19]–[22]. In addition, *Wolbachia* has also been shown to be required for oogenesis in the parasitoid *Asobara tabida* as its removal prevented the production of mature oocytes [23]. As a parasite, *Wolbachia* acts as a reproductive manipulator either by reducing the reproductive output of uninfected females via cytoplasmic incompatibility (CI), or by shifting the sex ratio of offspring in favour of females by either killing male offspring, inducing feminisation in genetic males or thelytokous parthenogenesis [12].

The whitefly, *Bemisia tabaci* (Gennadius) (Hemiptera: Aleyrodidae), is a cryptic species complex composed of numerous species [24]–[26]. All members of the complex are known to be infected with endosymbionts [27]–[30]. In China, *B. tabaci* was first recorded in 1949 [31], but it was not considered an important pest until the invasions by Middle East-Asia Minor 1 (commonly referred to as the B biotype, hereon MEAM1) and Mediterranean (commonly referred to as the Q biotype, hereon MED) [24]–[26]. As well as these two invaders, China is home to a number of indigenous members of the complex [26]. MEAM1 and MED now cause considerable damage to a wide range of crops in 30 of China’s 34 provinces [32]. In the last decade the endosymbiont community within *B. tabaci* has been studied extensively. Across the complex, one primary endosymbiont, *Portiera aleyrodidarum* as well as six secondary endosymbionts, *Arsenophonus*, *Cardinium*, *Fritschea*, *Hamiltonella*, *Rickettsia* and *Wolbachia* have been found in various populations [7], [27], [33]–[39]. In China, infection by *Wolbachia* in *B.* *tabaci* has been previously surveyed and reported. Ahmed et al. [28] collected 350 individuals from 29 different populations across China and the PCR detection revealed that 89.4% were infected, 33.6% with supergroup A, 50.9% with supergroup B, and 4.8% with both. In 2005–2008 Chu et al. [30] analysed 373 MEAM1 and 1830 MED individuals collected from 15 locations across 11 provinces. Infection in MEAM1 ranged from 5.9–36.4% while in MED ranged from 1.0–8.4%. Both studies indicated that *Wolbachia* prevalence varied considerably in regards to host plants, geographical location and whitefly species.

The primary endosymbiont provides its whitefly host with essential nutrients that cannot be obtained from the nutrient deficient phloem [33], while the function of the different secondary endosymbionts varies. Several of the secondary endosymbionts appear to affect the capacity of the host to be a pest. For example, *Hamiltonella* facilitates the transmission of plant viruses [40] while *Rickettsia* confers heat tolerance, increases susceptibility to some insecticides [29], [41] and also leads to the increased fecundity, greater survival to adulthood, faster development times and an increased proportion of daughters [42]. However, the full range of functions is still unknown and in many cases the perceived role is the result of correlative rather than experimental studies.

In order to identify the function of one endosymbiont in its arthropod host, two methods have been used. The first is through the use of microinjection to introduce a new endosymbiont and then make comparisons against an uninfected control [43]–[45], but this has not yet been applied to whiteflies. The second, undertaken for a number of different insect species, is through the elimination or inactivation of a specific endosymbiont, often through the use of selective antibiotics [46], [47]–[49]. However, very few studies have used the latter method for *B. tabaci* and/or its parasitoids. Ruan et al. [50] used three antibiotics (tetracycline, ampicillin trihydrate and rifampicin) to eliminate endosymbionts. They showed that any of the three antibiotics at 50 ug/ml and 48 h exposure, eliminated *Hamiltonella defensa*, *Wolbachia* and *Arsenophonus*, but failed to remove the primary endosymbiont *P. aleyrodidarum*. Ahmed et al. [27] compared the efficiencies of the same antibiotics in removing the secondary endosymbionts in three species of *B.* *tabaci*, Middle East- Asia Minor 1, Mediterranean and AsiaII_7 and showed differential responses between the antibiotics and the different species and their endosymbionts. The antibiotic rifampicin has also been used to remove *Rickettsia* and *Hamiltonella* from two aphelinid parasitoids, *Eretmocerus* sp. nr. *emiratus* and *Eretmocerus eremicus* [48].

Despite this research the physiological roles of *Wolbachia* in whitefly *B. tabaci* remains poorly defined. To better define the role of *Wolbachia* in *B. tabaci* we use an antibiotic to eliminate it selectively from its host and then compared the biology of infected and uninfected individuals. Furthermore, aphelinid parasitoids belonging to the genera *Encarsia* and *Eretmocerus* are important parasitoids of *B. tabaci* [51]. A rich complex of at least 19 species of aphelinid parasitizing *B. tabaci* has been reported from surveys across South China, among which *Encarsia bimaculata* and *Eretmocerus* sp. nr. *furuhashii* (Hymenoptera: Aphelinidae) are the most abundant [52], [53]. Previous research has shown that endosymbionts in the insect host may influence interactions with its parasitoid [43], [44], and we explored this for *B. tabaci* and the parasitoid *E. bimaculata*.

## Materials and Methods

### Plant Rearing

Cowpea, variety Yuefeng was chosen due to its economic value in South China. Seeds were grown in 1.3 L plastic pots containing a soil-sand mixture (40% sands, 5% clay and 55% peat) in a greenhouse under ambient conditions. Plants were watered as needed and were used when they were three weeks old and had 4–6 expanded true leaves.

### Insect Cultures

*Bemisia tabaci* MED was collected from eggplant in May of 2006 in Nantong, Jiangsu and then maintained in a glasshouse on hibiscus (*Hibiscus rosa-sinensis*) under ambient conditions. It was identified using mitochondrial cytochrome oxidase I [54] and then alignment against the consensus sequences determined by Dinsdale et al. [24].The parasitoid, *Encarsia bimaculata*, was collected from parasitized *B. tabaci* nymphs on hibiscus in Tianhe, Guangzhou; the species was identified by J. Huang (Fujian Agriculture and Forestry University, China). Voucher specimens have been deposited into the Insect Museum of the Department of Entomology, South China Agricultural University, Guangzhou. It is a solitary, arrhenotokous, heteronomous, autoparasitoid where the mated female lays female eggs internally into unparasitised nymphs whereas male eggs are placed into parasitized nymphs [55]. The parasitoid culture was maintained on *B. tabaci* feeding on hibiscus at 26.0±0.5°C, 70–80% relative humidity, 14∶10 (L:D) photoperiod, and a light intensity of approximately 3000 Lux.

### Detection of Endosymbionts

To extract DNA, individual whiteflies were first washed with double distilled water to remove alcohol and then homogenized in 200 µl lysis buffer (1% SDS, 10 mM Tris-HCl, pH 8.0, 25 mM EDTA, 25 mM NaCl, proteinase K 200 mg/ml) in a 0.5 ml microcentrifuge tube. The homogenate was incubated at 55°C for 2–3 h in a water bath and then at 95°C for 10 min to inactivate the proteinase K. After incubation, samples were centrifuged for 1 minute and then either used directly for PCR amplification or stored at −20°C for later use. The primers and PCR conditions used to detect the primary endosymbiont, *P. aleyrodidarum* and secondary endosymbionts, *Arsenophonus*, *Cardinium*, *Fritschea Hamiltonella*, *Rickettsia* and *Wolbachia* were from Zchori-Fein & Brown [33], Thao & Baumann [34], Everett et al. [35], Weeks & Breeuwer [56], Gottlieb et al. [57] and Li et al. [58] (Table S1).

For each of 150 individual whiteflies selected at random, PCR was undertaken using a 25 µl reaction volume and included: 2.5 mM MgCl_2_, 200 mM for each dNTPs, 1 µM of each primer, 1 unit DNA Taq polymerase (Invitrogen). Samples were amplified using a thermocycler (Bioer xpcycler, TC-XP-D). After amplification, 5 µl of the reaction mix was electrophoresed using 1.0% agarose gels with a DL2000 bp DNA marker in 1×TAE at 8 V/cm for 2 h; after electrophoresis the gel was then stained using 10 mg/ml ethidium bromide for 30 min. Bands were visualized using UV light with expected size were visible in the gels. Five products were sequenced for each endosymbiont using an ABI 3730XL automated DNA sequencer (PE Applied Biosystems). The resulting sequences were compared to the known sequences in GenBank using BLAST to verify their identity. The sequences of *P. aleyrodidarum* (AY429619), *Arsenophonus* (FJ766370), *Cardinium* (FJ766339), *Fritschea* (AY140910), *Hamiltonella* (JF795506), *Rickettsia* (JF795501) and *Wolbachia* (FJ545748) were used as the references for identification.

### The Inactivation of Endosymbionts with Rifampicin

To enable us to explore the possible roles of endosymbionts in *B. tabaci* it was necessary to have lines that were either infected or uninfected by a particular endosymbiont. The antibiotic rifampicin has been used to inactivate endosymbionts in whiteflies, but the level of reported inactivation varies across different studies [27], [50]. Artificial feeding through a parafilm membrane was used to deliver rifampicin to adult whiteflies so as to determine whether it could be used to selectively eliminate one or more species of endosymbiont. The method involved stretching three layers of parafilm (American National Can, Chicago, IL, USA) across the open end of a glass tube, 5 cm diameter and 10 cm height. The tube was then set upright on a tray in a controlled environment insect chamber with 26.0±0.5°C, 70–80% relative humidity, 14∶10 (L:D) photoperiod. The feeding solution containing 20% sucrose and 1.0 mg/ml rifampicin in double distilled water was injected between the bottom and middle layers of parafilm while a fresh cowpea leaf was inserted between the middle and upper layers so as to attract the whiteflies in the tube to the diet. Forty pairs of whitefly adults were released into the tube through the lower open end and were then allowed to feed for either 12, 24, 36 or 48 h. The control for each exposure period was the same as the above except that the feeding solution did not contain rifampicin. There were four replicates for each exposure period and control.

PCR, as described above, was followed by both fluorescence in situ hybridization (FISH) for *Wolbachia* and real-time PCR (qRT-PCR) for both the primary and secondary endosymbionts. FISH was used to confirm the results for *Wolbachia* elimination, while qRT-PCR was used to determine (a) whether time influenced inactivation and (b) which of the endosymbionts was inactivated by the antibiotic. Real-time PCR enables the activity of the endosymbionts to be measured as an inactive bacterium may still yield a positive result using PCR. For FISH, 10 whitefly pairs were selected at random from *Wolbachia* positive populations; five were then introduced into separate leaf cages to lay eggs. The eggs, except for 10, were left to develop to 3^rd^ instars. The 10 selected eggs were used for FISH detection of *Wolbachia*. The other 5 whitefly pairs were first treated with 1.0 mg/ml rifampicin for 48 h as above and were then also introduced into separate leaf cages for oviposition. All of the eggs, except for 10, were left to develop to 3^rd^ instars. Then the treated adults, 10 of their F1 eggs and 10 of the 3^rd^ instar nymphs were analysed for the presence of *Wolbachia* using FISH. The FISH procedure followed the method of Sakurai et al. [59] with slight modifications (Method S1). All the samples were observed using an inverted fluorescence microscope (Nikon Eclipse Ti-U). Specificity of the detection was confirmed using the *Wolbachia* negative whiteflies as the controls.

For qRT-PCR, total RNA from the whiteflies was extracted and the related cDNAs were then transcribed. Ten pairs of the whiteflies from each exposure period were collected at random and placed into a 0.5 ml microcentrifuge tube and then homogenized in 500 µl Trizol buffer. The total RNA was extracted using the Trizol RNA Extraction Kit (Omega) according to the method provided by the manufacturer. The RNA was then reverse-transcribed to cDNA using M-MLV (Invitrogen) and random hexamer primers (Invitrogen) as recommended by the manufacturer. qRT-PCR was performed using the cDNAs as targeted transcripts with QuantiTect primers in the CFX-96 PCR system (Applied Bio-Rad). The primers and qRT-PCR protocols are listed in Table S1, and the qRT-PCR analysis was repeated three times for each endosymbiont.

As *Wolbachia* was shown by the above to be the only endosymbiont inactivated by rifampicin under inactive conditions of 1.0 mg/ml for 48 h, we then explored whether the inactivation of *Wolbachia* influenced the biology of *B. tabaci* and parasitisation of *B. tabaci* by *E. bimaculata*.

### Effect of Rifampicin on the Biology of *B. tabaci*

Five pairs of adults from each of exposure period and their positive control (*Wolbachia* positive adults without inactivation) were collected at random from each of the four replicates and each pair then placed separately into one of five leaf cages on cowpea plants. As an additional negative control, 25 pairs of *Wolbachia* negative whitefly adults were selected at random and divided into 5 equal groups for the 0, 12, 24, 36 and 48 h exposures to rifampicin and released into leaf cages on cowpea plants as negative control experiment. Both the positive and negative controls were repeatedly screened using PCR throughout the culturing process to ensure that were either 100% infected or uninfected. The females were then allowed to lay eggs for 24 h after which the adults were removed, the eggs left to hatch and the nymphs to complete their development. All the experiments were undertaken at 26.0±0.5°C, 70–80% relative humidity and 14∶10 (L:D) photoperiod. There were four replicates. The size of the whitefly 4^th^ instar in each of the treatments and the controls was determined by first photographing the nymph (AxioCam, HRc and Coolsnap-Pro*cf* & CRI Micro^*^Color connected to a dissecting microscope, Discovery V20 Zeiss) and then measuring its length and width using Axio Vision Rel 4.8 software; 20 individuals from each treatment and the control were measured. The developmental time, survivorship, sex ratio and longevity of the F1 generation *B. tabaci* were determined.

### Effect of Rifampicin on Parasitisation by *E. bimaculata*

The 2^nd^-3^rd^ instar whiteflies from the 48 h inactivation treatment and control were prepared as above and then exposed to adult parasitoids. They were observed with the aid of a binocular microscope and when oviposition took place, the nymph was then marked. A total of 20 marked nymphs from the treatment and control were then selected at random and dissected to check for the presence of parasitoid eggs and their number. The parasitized nymphs that were not selected for dissection were left to allow the parasitoids to develop to pupa stage at which time 20 were selected at random from the treatment and controls. Each parasitoid pupa was then dissected from the whitefly host, photographed and the length and width of the head capsule then measured using the same methodology described above for the whitefly nymphs. The size of the head capsule is a reliable measure of parasitoid size.

In a parallel experiment, 20 pairs of adult *B. tabaci* were selected at random, divided into 5 equal groups and then treated with 1.0 mg/ml rifampicin for 0,12, 24, 36 and 48 h, respectively. In addition to the treated whiteflies, 20 pairs of *Wolbachia* positive and 20 pairs of *Wolbachia* negative whitefly adults were selected at random. Each adult pair was then introduced into a leaf cage on a cowpea plant and allowed to oviposit for 24 h. Once all the eggs had hatched in one leaf cage, all nymphs except 10 at the 2^nd^–3^rd^ instar stage were removed and a single mated *E. bimaculata* female (3–4 day old) was then released into the cage for 24 h and allowed to parasitize the nymphs. The wasp was observed ovipositing with the aid of a binocular microscope and the position of each nymph into which an egg was placed was then marked. When the F1 generation of *E. bimaculata* was visible (the first visible stage is the 2^nd^ instar which appears as a characteristic ‘C’ shape) in a whitefly nymph, the survivorship of parasitoid F1 juveniles (from egg to 2^nd^ instar, and from 2^nd^ instar to adult) was then calculated based on the numbers of eggs, visible 2^nd^ instars and the numbers of emerged adults. Additionally, 20 newly emerged *E. bimaculata* adults from each of the treatments as well as the controls were selected at random and released individually into one of 20 leaf cages with an abundance of eggs and 1^st^ to 3^rd^ instars. The parasitoids were observed daily until death so as to determine adult longevity. All the experiments were undertaken at 26.0±0.5°C, 70–80% relative humidity, 14∶10 (L:D) photoperiod, and each experiment was repeated four times.

### Data Analysis

To confirm whether inactivation of each of the endosymbionts had occurred, the expression levels of the different target genes were compared between the control and treatments and calculated using the Bio-Rad CFX software. Differences in developmental times, survivorship of whitefly and parasitoids juveniles, the body size of whitefly 4^th^ instar and the sex ratio and longevity of the whitefly and parasitoid adults were analysed using analysis of variance (multiple comparisons, PROC ANOVA, SAS Institute 2003) [60]; the differences in the head capsule size of *E. bimaculata* pupa and in the number of eggs that were oviposited between the control and the 48 h exposure treatment were analysed using a t-test (SAS Institute 2003) [60]. The normality and homoscedasticity of the percentage survivorship and adult sex ratio data were first tested and arcsine transformed before the analysis of variance. Means were separated using Tukey’s Studentized range (HSD) test at a significant level of a = 0.05 (SAS Institute 2003) [60].

## Results

### The Detection of Endosymbionts in the MED *B. tabaci*

PCR detection indicated that all seven endosymbionts, the primary obligate endosymbiont *P. aleyrodidarum* and the six secondary endosymbionts *Arsenophonus*, *Cardinium*, *Fritschea*, *Hamiltonella*, *Rickettsia* as well as *Wolbachia* were present in the populations sampled. All products were sequenced to confirm identity (GenBank accession numbers JQ009297 to JQ009303). All individuals were infected with the primary endosymbiont. The number of different endosymbionts in any one individual whitefly varied. *Wolbachia* was the most prevalent (78.6%) followed by *Arsenophonus* (59.4%), *Rickettsia* (35.5%), *Hamiltonella* (17.2%), *Cardinium* (13.7%) and *Fritschea* (8.3%) (Table 1); the identity of each was confirmed through sequencing of the amplicon. Of the individuals screened, 14.7% were uninfected with secondary endosymbionts while 10% of individuals were infected with *Wolbachia* only. No other secondary endosymbionts were present as single infections. 38.7% were infected by two endosymbionts, 21.3% with three and 15.3% with four (Table 1). No individuals were infected with more than four secondary endosymbionts (Table 1). Most, 91.2%, had an infection with *Wolbachia* plus at least one other secondary endosymbiont. Only 6.7% involved infections with secondary endosymbionts that were not *Wolbachia* (Table 1). The full details are presented in Table 1.

**Table 1:** The percentage infections by *Wolbachia*, *Arsenophonus*, *Rickettsia*, *Hamiltonella*, *Cardinium* and *Fritschea* in a sample of 150 individuals.

Endosymbiont code	Endosymbiont combinations	Percentage	Individuals
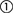	*Wolbachia*	78.6	118
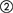	*Arsenophonus*	59.4	89
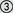	*Rickettsia*	35.5	53
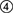	*Hamiltonella*	17.2	26
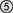	*Cardinium*	13.7	21
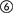	*Fritschea*	8.3	12
	nil infection		22
	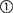		15
	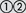		38
	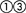		7
	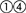		1
	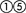		2
	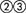		4
	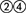		1
	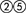		1
	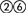		1
	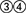		2
	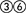		1
	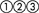		13
	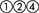		4
	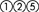		3
	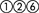		1
	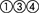		5
	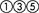		4
	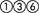		1
	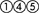		1
	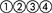		7
	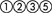		5
	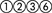		4
	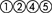		3
	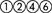		2
	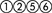		2
		n	150

The combinations of different secondary endosymbiont infections detected and the number of individuals in which each combination was present are also shown. The endosymbiont codes 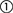 to 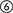 are used to identify the respective endosymbionts.

### The Inactivation of Various Endosymbionts with Rifampicin

PCR amplification indicated that only *Wolbachia* was eliminated after 48 h exposure to rifampicin (figure not shown). FISH analysis indicated that exposure to 1.0 mg/ml rifampicin for 48 h, eliminated *Wolbachia* from whitefly adults and their progeny (eggs and nymphs) (Fig. S1). The qRT-PCR gene expression for the primary endosymbiont, *P. aleyrodidarum* and the six secondary endosymbionts are shown in Fig. S2A–G, which also confirmed the initial PCR based detection in that the primary endosymbiont and each of secondary endosymbionts, except *Wolbachia*, were positive for each duration of exposure, and the expression levels of the target genes (16S/23S rDNA) were not significantly different regardless of the duration of exposure to rifampicin (Fig. S2 A–F). In contrast, while positive for exposure durations up to and including 36 h, *Wolbachia* was negative after 48 h exposure indicating there was no activity (Fig. S2G).

### Effects of *Wolbachia* Inactivation on the Development of *B. tabaci* Juveniles

**Figure 1:**
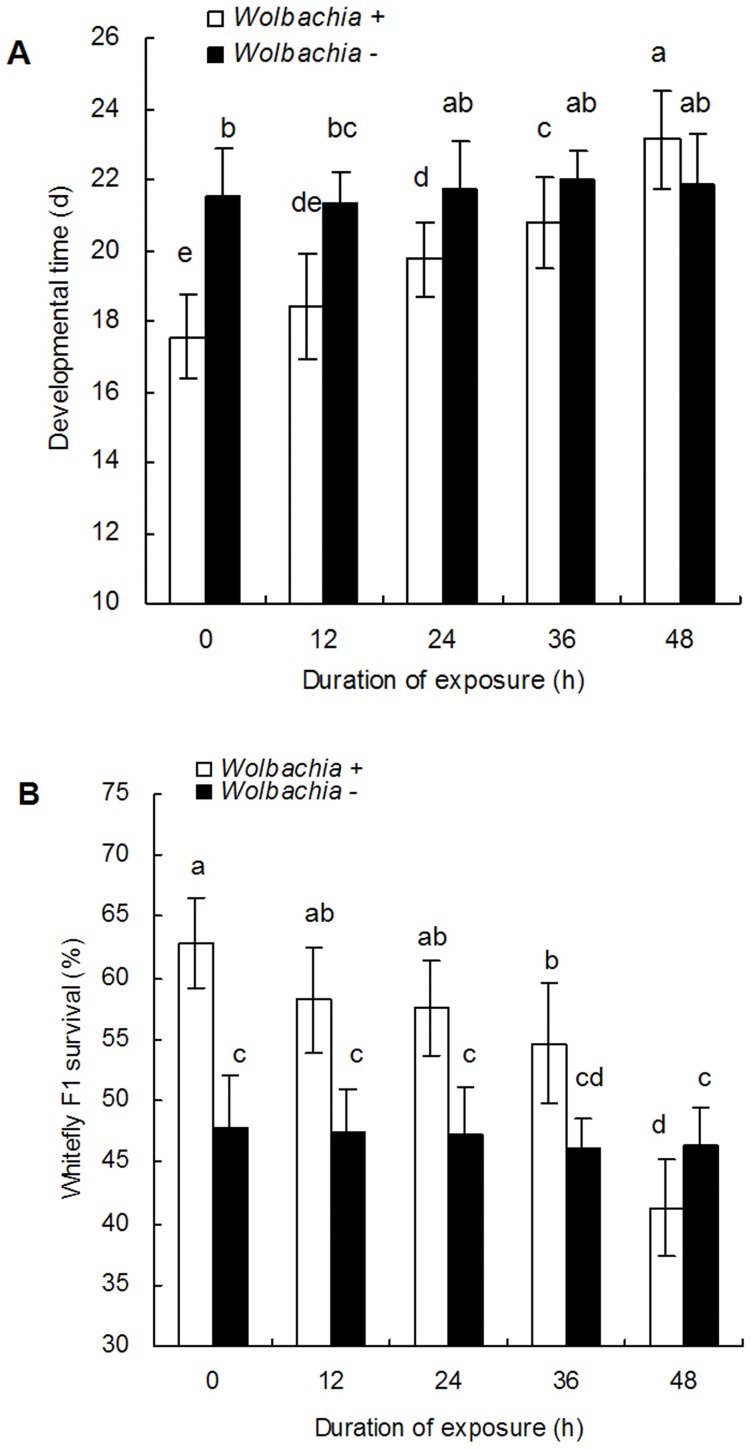
The development time (M±SE) and survival of the F1 progeny of *Wolbachia* positive (+) and negative (−) whitefly adults after different rifampicin treatments A: development time, B: survival. The exposed time to 1.0 mg/ml rifampicin is either 12, 24, 36 or 48 h. 0 is the untreated control. The different letters indicate a significant difference using Tukey HSD.

Compared with the mean development duration of 17.6±1.2 days in the *Wolbachia* positive controls, the developmental time of the F1 progeny increased the longer the parents were exposed to rifampicin (Fig. 1A, F_9,190_ = 22.4, P<0.0001). Similarly, the mean percentage of F1 juveniles that completed their development declined as exposure to rifampicin in the parents increased (Fig. 1B, F_9,190_ = 28.4, P<0.0001). All exposure durations lead to significant declines in the percentage of nymphs that completed their development relative to the untreated control with the lowest percentage survival, 41.3±2.2%, being observed after 48 h exposure. On the other hand, the mean developmental time of the F1 progeny from *Wolbachia* negative whitefly adults was longer (21.3 to 22.0 days) and mean survivorship lower (46.2% to 47.9%) than that observed in the *Wolbachia* infected treatment, but no significant differences were found between different exposure periods (Fig. 1A, B).

### Effects of *Wolbachia* Inactivation on the Sex Ratio and Longevity of *B. tabaci* Adults

**Figure 2:**
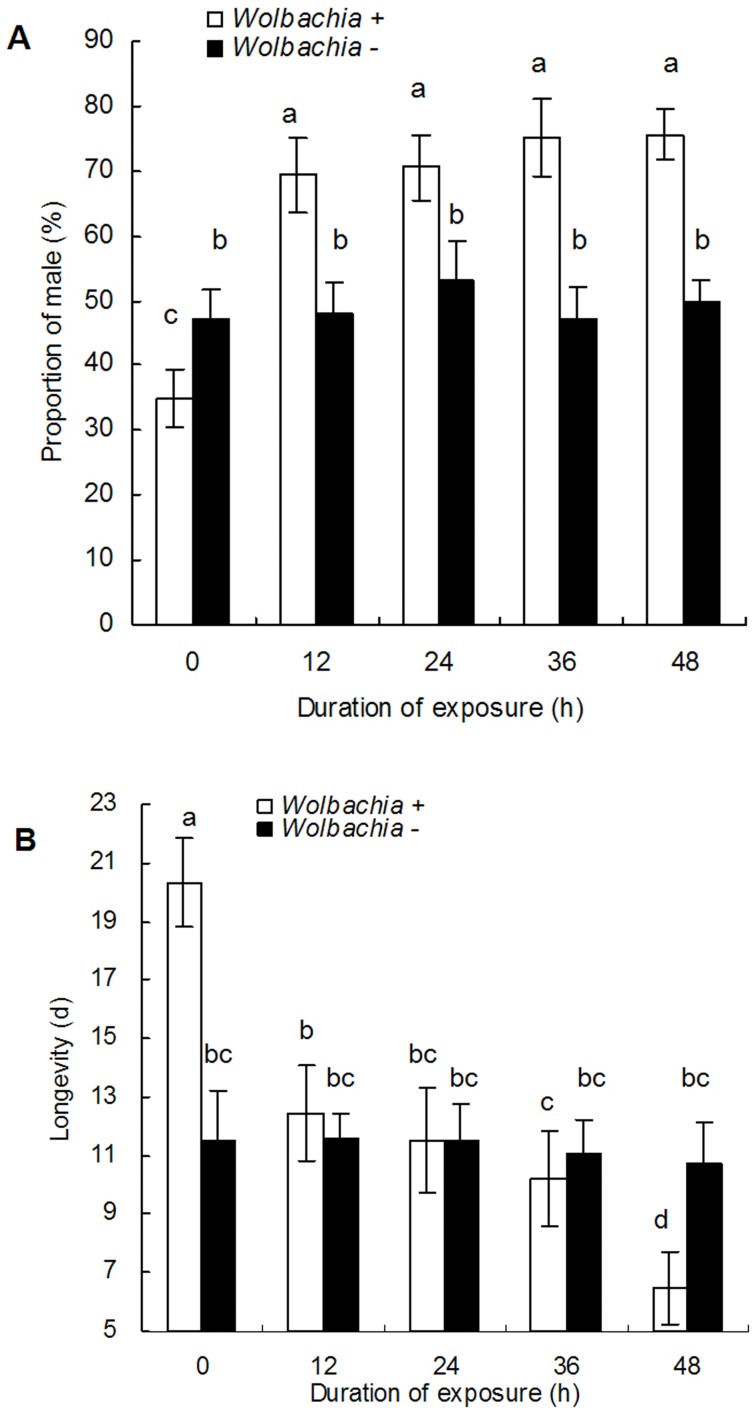
The averaged longevity (M±SE) of the F1 adults and the proportion of male F1 progeny from whitefly adults. A: male proportion, B:longevity. The exposed time to 1.0 mg/ml rifampicin is either 12, 24, 36 or 48 h. 0 is the untreated control. The different letters indicate a significant difference using Tukey HSD.

As the duration of adult exposure to rifampicin increased, the mean percentage of male progeny increased from an average of 35.0±2.5% in the untreated control to 75.6±5.3% after 48 h of exposure (Fig. 2A, F_9,190_ = 85.7, P<0.0001), but there was no significant differences among the F1 progeny from various antibiotic exposures. The duration of exposure to rifampicin was also associated with a significant decline in the average longevity of F1 adults (Fig. 2B, F_9,190_ = 55.5, P<0.0001), with the lowest longevity resulting occurring after 48 h of exposure in the parents. However, results also indicated that different antibiotic treatments had no significant effects on either the proportion F1 males or the longevity of F1 adults from *Wolbachia* negative whitefly parents (Fig. 2A, B).

### Effects of *Wolbachia* Inactivation on the Body Size of Whitefly and Head Capsule Size of the Parasitoid

Inactivation of *Wolbachia* significantly affected the development of both the whitefly nymph and the parasitoid. The length of whitefly 4^th^ instar declined as exposure to rifampicin in the parent increased (F_4,95_ = 79.9, P<0.0001, Table 2,) as did the width (F_4,95_ = 128.9, P<0.0001, Table 2, Fig. S3). In particular, the length in the control was 0.81±0.01 mm which was 39.5% longer than that observed after 48 h exposure, 0.58±0.01 mm. Similarly, the width of the 4^th^ instar in the control was 0.63±0.01 mm which was 76.7% wider than that observed after 48 h exposure to the antibiotic, 0.36±0.01 mm (Table 2). In the case of the parasitoid, the head capsule in the control was significantly wider (43.4%, T = 9.4, P<0.0001) and longer (21.7%, T = 9.5, P<0.0001) (Table 3, Fig. S4) than those pupae removed from nymphs that lacked a *Wolbachia* infection.

**Table 2:** The length and width (±SE) of whitefly 4^th^ instars from parents exposed to 1.0 mg/ml rifampicin for 12, 24, 36 or 48 h; 0 is the untreated control.

	Duration of exposure to rifampicin (h)					
Size(mm)	0	12	24	36	48	
Length	0.81±0.01 a	0.75±0.01 a	0.71±0.01b	0.64±0.0 b	0.58±0.01 c	F_4,95_ = 79.9, p<0.0001
Width	0.63±0.01 a	0.59±0.01 a	0.56±0.01 a	0.48±0.01 b	0.36±0.01 c	F_4,95_ = 128.9, p<0.0001

**Table 3:** Comparison of the length and width of the head capsule (±SE) of *Encarsia bimaculata* pupa from the untreated control (0 h) with pupa after parental exposure for 48 h to 1.0 mg/ml rifampicin.

	Duration of exposure (h)		
Size(mm)	0	48	
Length	0.12±0.002	0.08±0.003	t = 9.4, p<0.0001
Width	0.23±0.003	0.19±0.003	t = 9.5, p<0.0001

### Effects of *Wolbachia* Inactivation on the Development and Survival of *E. bimaculata* Juveniles

**Figure 3:**
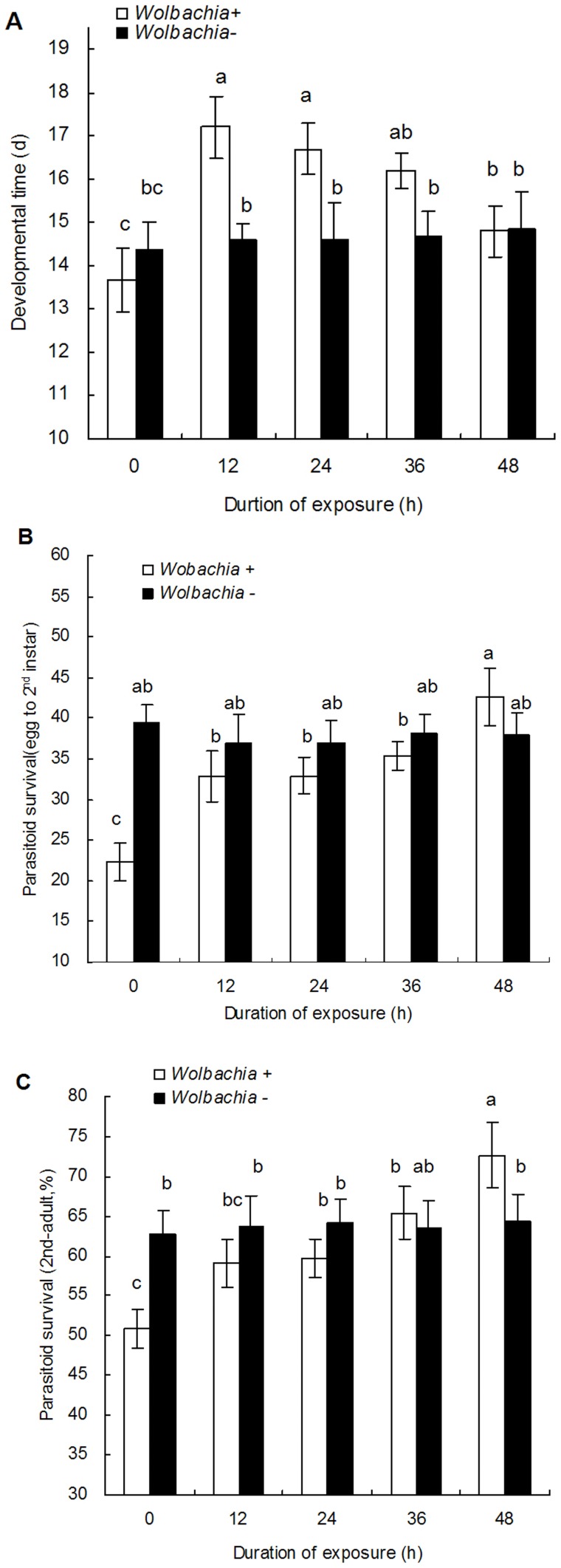
The developmental time and survival (M±SE) of *E. bimaculata* in F1 *B. tabaci* nymphs from different rifampicin treatments. A: developmental time, B: survival of egg to 2^nd^ instar larvae, C: survival of 2^nd^ instar larvae to adults. The exposed time to 1.0 mg/ml rifampicin is either 12, 24, 36 or 48 h. 0 is the untreated control. The different letters indicate a significant difference using Tukey HSD.

*Wolbachia* inactivation in *B. tabaci* also had significant effects on the development and survival of the parasitoid. The development time of *E. bimaculata* was significantly longer in the nymphs from parents which had been exposed to rifampicin (Fig. 3A, F_9,190_ = 11.8, P<0.0001) for either 12, 24 or 36 h. Here, the mean developmental time of the untreated whiteflies was 13.7±0.7 days, while for the nymphs from parents exposed to rifampicin for either 12, 24 or 36 was 17.2±0.7, 16.7±0.6 and 16.2±0.4 days, respectively. However, in the nymphs in which *Wolbachia* had been completely inactivated, the development time of 14.8±0.6 days was not significantly different to that observed in the untreated nymphs.

Dissection of the nymphs after oviposition showed that in each case at least one egg was deposited. In the case of the control, 19 nymphs had one egg present and the remaining nymph had two whereas in the nymphs where *Wolbachia* had been eliminated, all parasitized nymphs had a single egg present. There were no significantly differences in the number of eggs oviposited by *E. bimaculata* in nymphs positive for *Wolbachia* and those that were negative.

As for the survival, both the survivorship of *E. bimaculata* from egg to 2^nd^ instar and 2^nd^ instar to adult from parents exposed to rifampicin increased with the increasing duration of exposure to the antibiotic (Fig. 3B, F_9,190_ = 23.9, P<0.0001; Fig. 3C, F_9,190_ = 9.55, P<0.0001). The overall survivorship of *E. bimaculata* in the F1 whitefly progeny that were not infected with *Wolbachia* (48 h exposure), was significantly higher than that observed following shorter periods of exposure or no exposure at all. Most of the mortality was observed in the period from egg to 2^nd^ instar. In contrast, the development time of *E. bimaculata* in F1 progeny from *Wolbachia* negative whiteflies was significantly shorter (14.35±0.67 to14.85±0.87 days) and survivorship significantly higher for both egg-2^nd^ instar (36.97±2.74 to 39.49±2.09%) and 2^nd^ instar to adult (62.68±2.99 to 64.38±3.44%) than those parasitoids that developed from *Wolbachia* infected hosts, but no significant differences were found between the different antibiotic exposures for each set of treatments.

### Effects of *Wolbachia* Inactivation on the Longevity of *E. bimaculata*

**Figure 4:**
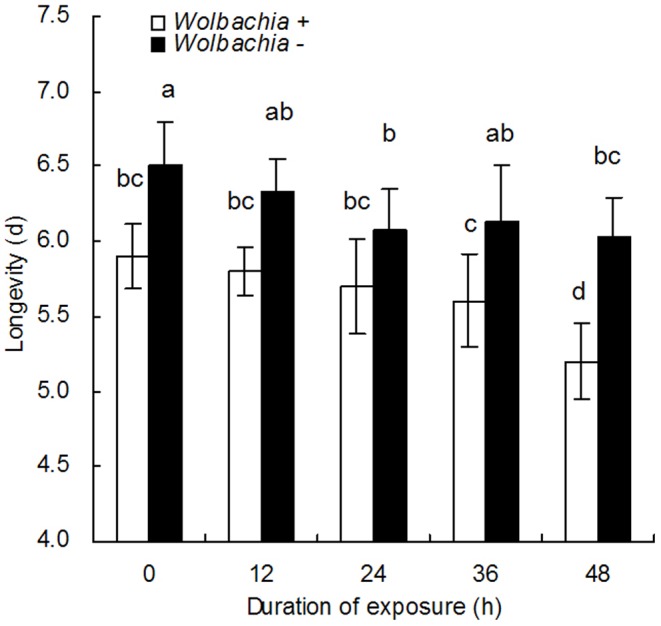
The longevity (M±SE) of *E. bimaculata* female adults developing in F1 *B. tabaci* nymphs. The whitefly nymphs from parents exposed to 1.0 mg/ml rifampicin for either 12, 24, 36 or 48 h. 0 is the untreated control. The different letters indicate a significant difference using Tukey HSD.

The longevity of *E. bimaculata* females that emerged from the nymphs is shown in Fig. 4. There was no significant difference between the longevity of parasitoids from positive control nymphs, 5.9±0.4 days, and the longevities of parasitoids exposed to rifampicin for 12, 24 and 36 h, here longevities were 5.8±0.3, 5.7±0.3, 5.6±0.2 days, respectively. However, the longevity of parasitoids that emerged from nymphs where the parents had been exposed to rifampicin for 48 h was significantly shorter, 5.2±0.12 days (F_9,190_ = 14.2, P<0.0001). In this treatment *Wolbachia* was completely inactivated which indicates that the presence of *Wolbachia* significantly reduces the longevity of these parasitoids. Furthermore, parasitoids that develop in the progeny from *Wolbachia* negative females were significantly longer than that observed for parasitoids that developed in *Wolbachia* positive nymphs.

## Discussion

Symbiotic associations between bacteria and insects are common in nature [48], but disentangling the roles of different endosymbionts is challenging when one host contains more than one endosymbiont and so studies that selectively remove an endosymbiont are one way to infer the roles played by different endosymbionts. While FISH is usually used to confirm the elimination of the endosymbiont [29], [49], [61], [62], we found that qRT-PCR can be used to determine the level of activity of *Wolbachia*. We used both and through this approach showed that exposure to 1.0 mg/ml rifampicin for 48 h completely inactivated *Wolbachia* in *B. tabaci* without reducing the activity of either the primary or non-*Wolbachia* secondary endosymbionts. We also show that the duration of exposure was critical as exposure times shorter than 48 h meant that some *Wolbachia* activity remained.

The biological contribution of bacteria endosymbionts to their hosts have been studied in detail [11], [12], [29], [43], [63]–[68], but the experimental evidence for the biological and ecological roles of *Wolbachia* in many of its insect hosts has remained largely unexplored [14], [46] with perhaps the exception of studies on host reproduction (reviewed in [69]–[72]). In addition to host reproduction, Fytrou et al. [46] observed that the presence of *Wolbachia* was associated with significant increases in the susceptibility of *D. simulans* to parasitisation by *L. hererotoma*. Here, *Wolbachia* infected *D. simulans* were less able to encapsulate parasitoid eggs, compared to those free of infection. Here, the removal of *Wolbachia* from the parasitoid was beneficial as its eggs suffered significantly lower encapsulation rates. In the case of the bedbug, *Cimex lectularius*, infections with *Wolbachia* increased both the rate of growth and egg viability. Furthermore, the negative effects associated with the removal of *Wolbachia* could be reversed by oral administration of B vitamins indicating that *Wolbachia* was also associated with the synthesis of essential non-dietary nutrients [14]. *Wolbachia* is also known to protect *Drosophila melanogaster* from infections of the *Drosophila* C viruses [19], [20].

In our study we showed that in MED, *Wolbachia* decreased the development time of the nymphs, increased the likelihood that nymphs completed their development, increased the life span of the adult whitefly and increased the proportion of progeny that developed into females. These indicate that the *Wolbachia* infection increases the fitness of the whitefly relative to its uninfected competitors. The faster development, higher survival and larger body size of the infected 4^th^ instar relative to the uninfected nymphs suggests that *Wolbachia* may confer some nutritional and immune benefits on the host, however, the physiological roles of *Wolbachia* may vary in other *B. tabaci* species since its phenotypic effects can be affected by the genotype of its host. More work is needed on this and the overall ecological roles and interactions played by *Wolbachia* as well as the other secondary endosymbionts.

The reduction in nymph body size also appears to have had several consequences for the parasitoid. Firstly, there was a complex interaction between duration of exposure to the antibiotic and parasitoid development time. Initially, shorter parental exposures (12 to 36 h) to rifampicin lead to a marked increase in the time taken for the parasitoid to complete development in the F1 nymphs. The developmental time of the whitefly increased along with the increasing duration of exposure whereas the developmental time of the parasitoid decreased. These opposing responses maybe due to the nonlinear effects of *Wolbachia* inactivation in the two different trophic levels. This is not unexpected as cause-and-effect relationships in ecological food-webs are often nonlinear [73]–[76]. In the case of the whitefly, the removal of *Wolbachia* has a direct effect on the host whereas the removal in the whitefly is an indirect effect in regards to the parasitoid. Alternatively, the slow-growth, high-mortality hypothesis suggests that the shorter development time of *E. bimaculata* in smaller and therefore lower quality hosts, may reduce the risk of not completing development [77]. The acceleration of development on small hosts is a common phenomenon in parasitoids [78]. Furthermore, as in our case, Sequeira & Mackauer [79], [80] observed an initial slowing of the rate of development on intermediate sized hosts followed by a marked increase on the smallest hosts.

Secondly, the survival of parasitoid larvae increased as the duration of parental whitefly exposure to rifampicin increased, i.e. the percentage of parasitoids that completed their development in whitefly nymphs that lacked *Wolbachia* was considerably higher than that observed in infected nymphs. However, the longevity of the adult parasitoids emerging from nymphs lacking *Wolbachia* was considerably shorter than that observed for adults emerging from infected nymphs. This reduced longevity was also associated with the observation that the parasitoids developing in nymphs free of infection, were significantly smaller. The relationship between host size and size of the emerging parasitoid is also well known and it is not unexpected that a small host will lead to a small parasitoid adult with reduced fitness [81], [82]. Finally, while there was no difference in the number of eggs oviposited into infected and uninfected nymphs, parasitoid eggs inserted into nymphs that were uninfected by *Wolbachia* were more likely to complete their development than those inserted into infected nymphs suggesting that *Wolbachia* provided some protection against parasitisation.

In summary, our findings showed that *Wolbachia* is involved in many aspects of whitefly host biology including development duration, reproduction and defense. Our results lead us to speculate that *Wolbachia* may play a role in nutrient supplementation as its removal was associated with a decline in nymph body size and not surprisingly, smaller nymphs resulted in smaller, less fit parasitoids. We know from previous research [14] that *Wolbachia* can play this role and so more work is needed here to determine whether this is the case. *Wolbachia* also plays a protective role as eggs inserted into infected nymphs were less likely to complete development. Finally, *Wolbachia* also plays a role in the manipulation of whitefly sex ratio as a greater proportion of female progeny are produced by infected rather than uninfected females. Our observations therefore suggest that *Wolbachia* in *B. tabaci* is playing a role similar to that observed in other species.

Our results show that the elimination of *Wolbachia* in whitefly can result in delayed development, increased mortality, shortened lifespan, weakened ability to counter parasitism, and potentially, a slower rate of population increase due to the bias towards the production of male offspring. These suggest that novel technologies that target endosymbionts may provide new tools with which to manage these pests. However, much more research is needed to unravel the various and complex interactions that occur between the host, its endosymbionts and the environment before such tools are likely to provide effective control options.

## Supporting Information

Figure S1 **Fluorescence** ***in situ*** **hybridization of** ***B. tabac*** **i using a** ***Wolbachia*** **specific probe (red).** A: FISH of *Wolbachia* positive *B. tabaci* adult (a, b), its egg (c, d) and 3^rd^ nymph (e, f). The left panels are under natural light, the right panels under fluorescence. *Wolbachia* was found in the main body of the adult, egg and nymph. B: FISH of *Wolbachia* positive *B. tabaci* adult (a, b), its egg (c, d) and nymph (e, f) after 48 exposure to 1.0 mg/ml rifampicin. The left panels are under natural light, the right panels are under fluorescence. The absence of red fluorescence showed that *Wolbachia* can be completely eliminated in the *B. tabaci* adult, egg and third instar.
(TIF)

Figure S2 **The relative expression levels of target genes used to measure activity in each of the different endosymbionts in the qRT-PCR.** 0 is the untreated control and 12, 24, 36 and 48 h indicate the duration of exposure to 1.0 mg/ml rifampicin. The lower the level of expression the greater the level of inactivation. A–G were *P. aleyrodidarum*, *Arsenophonus*, *Cardinium*, *Fritschea*, *Hamiltonella*, *Rickettsia* and *Wolbachia*, respectively.
(DOC)

Figure S3 **The body size of the** ***Bemisia tabaci*** **4^th^ instar.** 0 is the untreated control and 12, 24, 36 and 48 h indicate the duration of exposure to 1.0 mg/ml rifampicin. The body size was first photographing using a microscope (Discovery V20 Zeiss) connected to a camera (AxioCam, HRc and Coolsnap-Pro*cf* & CRI Micro^*^Color) and then measured using Axio Vision Rel 4.8 software.
(TIF)

Figure S4 **Head capsule size of** ***Encarsia bimaculata*** **pupa.** The wasps developed from either whitefly nymphs infected with *Wolbachia* or those that were uninfected as a result of 48 h exposure to 1.0 mg/ml rifampicin. The head size was first photographing using a microscope (Discovery V20 Zeiss) connected to a camera (AxioCam, HRc and Coolsnap-Pro*cf* & CRI Micro^*^Color) and then measured using Axio Vision Rel 4.8 software.
(TIF)

Table S1 **The primers and PCR programs used for the detection of the various endosymbionts.** Protocol-1 refers to standard PCR detection; Protocol-2 refers to the measurement of target gene expression using qRT-PCR.
(DOC)

Method S1 **Fluorescence** ***in situ*** **hybridization of** ***Bemisia tabaci*** **samples.**
(DOC)
