# Supplementary figures and images for: Inactivation of Wolbachia Reveals Its Biological Roles in Whitefly Host
Source: PLoS One. 2012 Oct 29;7(10):e48148. doi: 10.1371/journal.pone.0048148 (PMC3483251; doi:10.1371/journal.pone.0048148)

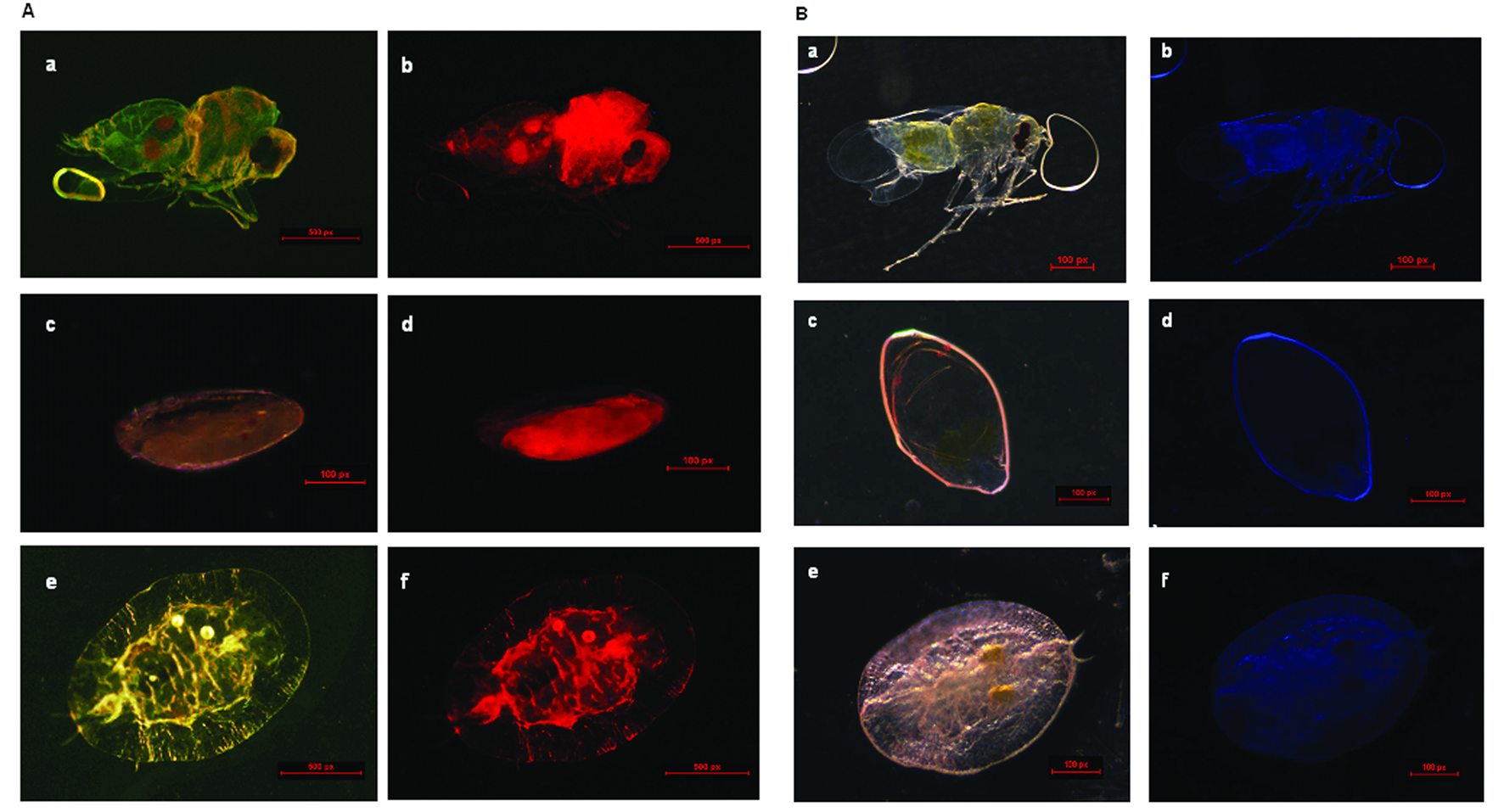

Supplement: Figure S1 — Fluorescence in situ hybridization of B. tabac i using a Wolbachia specific probe (red). A: FISH of Wolbachia positive B. tabaci adult (a, b), its egg (c, d) and 3rd nymph (e, f). The left panels are under natural light, the right panels under fluorescence. Wolbachia was found in the main body of the adult, egg and nymph. B: FISH of Wolbachia positive B. tabaci adult (a, b), its egg (c, d) and nymph (e, f) after 48 exposure to 1.0 mg/ml rifampicin. The left panels are under natural light, the right panels are under fluorescence. The absence of red fluorescence showed that Wolbachia can be completely eliminated in the B. tabaci adult, egg and third instar. (TIF) [file pone.0048148.s001.tif]

| **Normalized fold expression**  **A** | 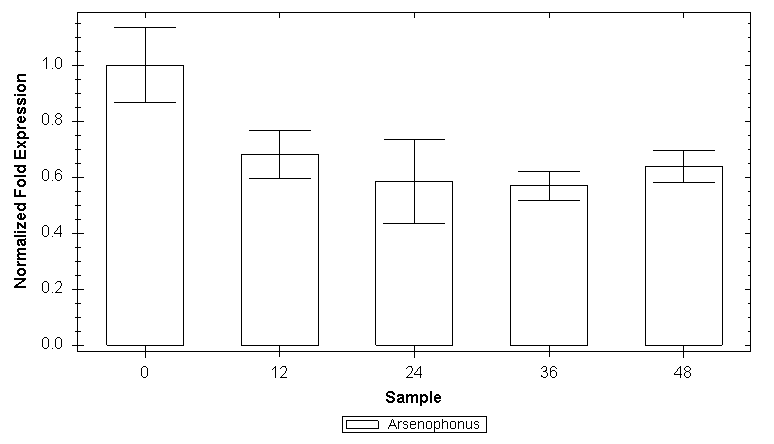  **B** |
| --- | --- |
| 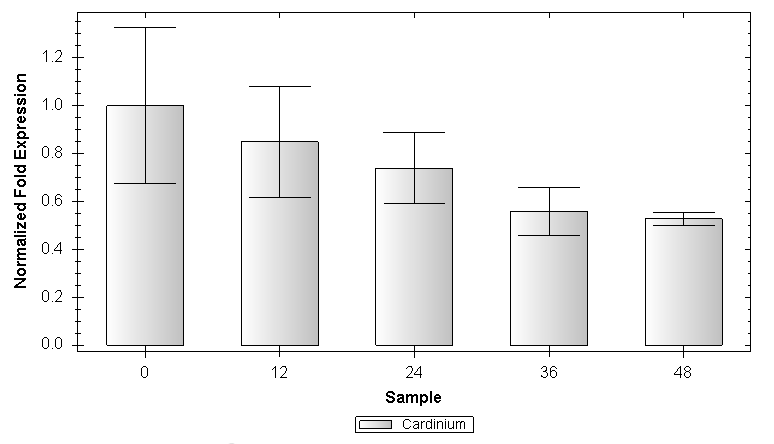  **C** | 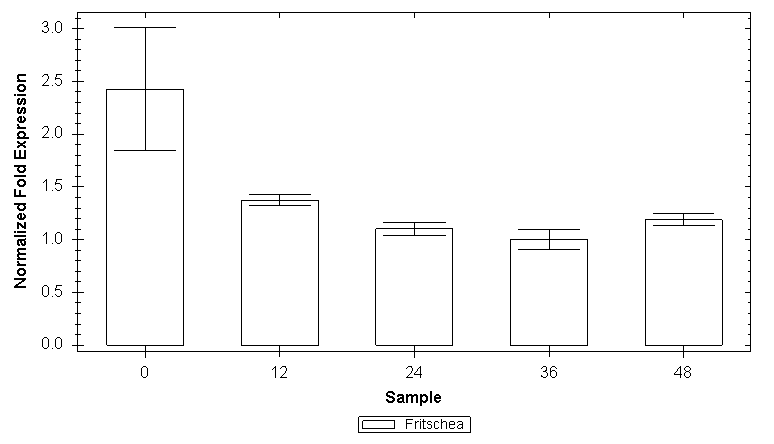  **D** |
| 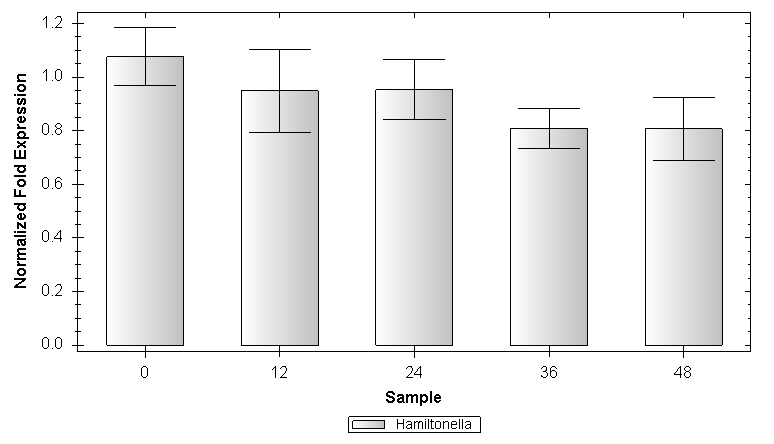  **E** | 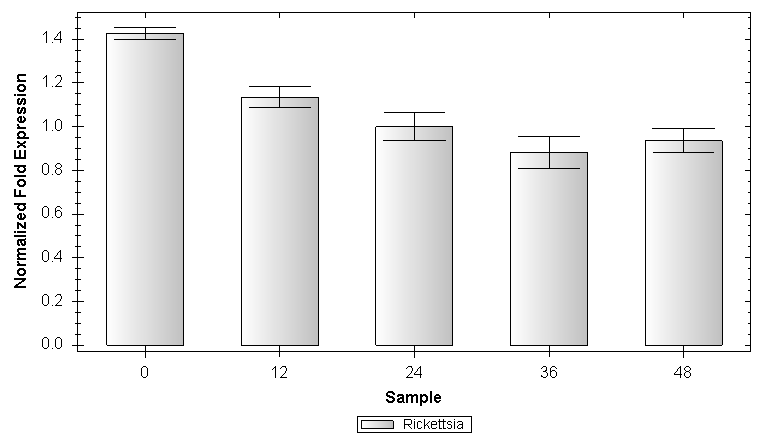  **F** |
| 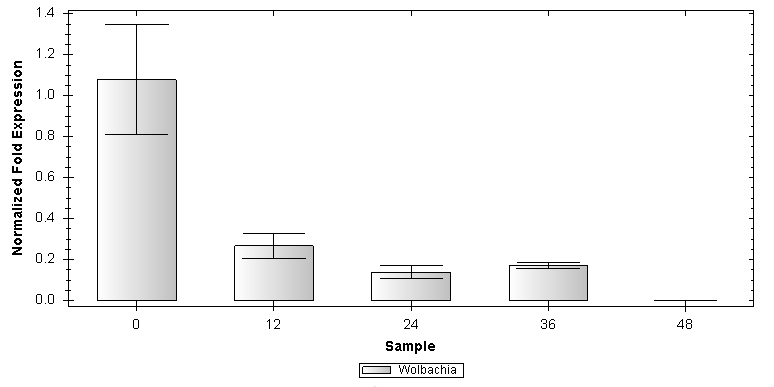  **G** |  |

**Duration of exposure (h)**

Supplement: Figure S2 — The relative expression levels of target genes used to measure activity in each of the different endosymbionts in the qRT-PCR. 0 is the untreated control and 12, 24, 36 and 48 h indicate the duration of exposure to 1.0 mg/ml rifampicin. The lower the level of expression the greater the level of inactivation. A–G were P. aleyrodidarum, Arsenophonus, Cardinium, Fritschea, Hamiltonella, Rickettsia and Wolbachia, respectively. (DOC) [file pone.0048148.s002.doc]

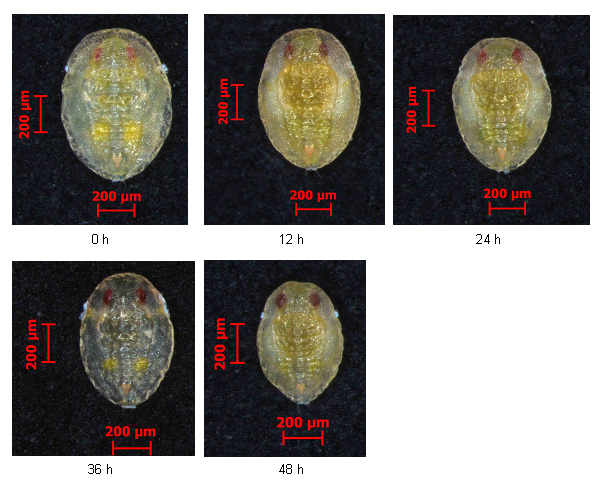

Supplement: Figure S3 — The body size of the Bemisia tabaci 4th instar. 0 is the untreated control and 12, 24, 36 and 48 h indicate the duration of exposure to 1.0 mg/ml rifampicin. The body size was first photographing using a microscope (Discovery V20 Zeiss) connected to a camera (AxioCam, HRc and Coolsnap-Procf & CRI Micro*Color) and then measured using Axio Vision Rel 4.8 software. (TIF) [file pone.0048148.s003.tif]

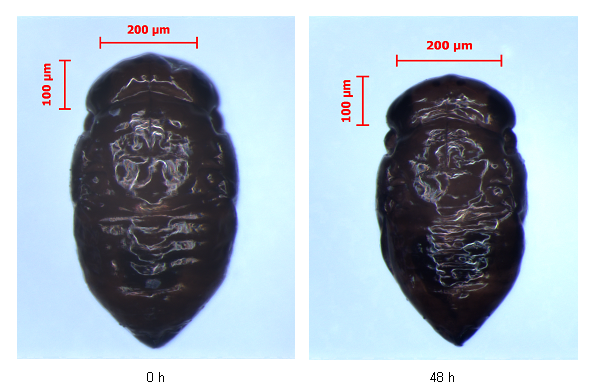

Supplement: Figure S4 — Head capsule size of Encarsia bimaculata pupa. The wasps developed from either whitefly nymphs infected with Wolbachia or those that were uninfected as a result of 48 h exposure to 1.0 mg/ml rifampicin. The head size was first photographing using a microscope (Discovery V20 Zeiss) connected to a camera (AxioCam, HRc and Coolsnap-Procf & CRI Micro*Color) and then measured using Axio Vision Rel 4.8 software. (TIF) [file pone.0048148.s004.tif]
