# Supplementary material for: Inactivation of Wolbachia Reveals Its Biological Roles in Whitefly Host
Source: PLoS One. 2012 Oct 29;7(10):e48148. doi: 10.1371/journal.pone.0048148 (PMC3483251; doi:10.1371/journal.pone.0048148)
Supplement: Table S1 — The primers and PCR programs used for the detection of the various endosymbionts. Protocol-1 refers to standard PCR detection; Protocol-2 refers to the measurement of target gene expression using qRT-PCR. (DOC) [file pone.0048148.s005.doc]

Table S1 The primers and PCR programs used for the detection of various endosymbionts. Protocol-1 refers to standard PCR detection; Protocol-2 refers to the measurement of target gene expression using qRT-PCR.

| Endosymbinot | Target gene |  | Primer and protocol | Primer references |
| --- | --- | --- | --- | --- |
| *Protiera aleyrodidarum* | 16S rDNA | Primer | 28F:5'-TGCAAGTCGAGCGGCATCAT-3'; 1098R:5'-AAAGTrCCCGCCTYATGCGT-3' | [33] |
|  |  | Protocol-1 | 95.0℃2min, then 94.0℃30s, 48.5℃30s, 72.0℃2min in 35 cycles, 72.0℃4min |  |
|  |  | Protocol-2 | 95.0℃3min, then 95.0℃10s, 50.0℃30s 72.0℃25s in 39 cycles, 72.0℃30s |  |
| *Arsenophonus* | 23S rDNA | Primer | Ars23S-F:5'-CGTI'rGATGAATI'CATAGTCAAA-3';Ars23S-R: 5'-GGTCCTCCAGTrAGTGTI'ACCCAAC-3' | [34] |
|  |  | Protocol-1 | 95.0℃2min, then 94.0℃30s, 50.0℃45s, 72.0℃2min in 35 cycles, 72.0℃4min |  |
|  |  | Protocol-2 | 95.0℃3min, then 95.0℃10s, 46.5℃30s, 72.0℃25s in 39 cycles, 72.0℃30s |  |
| *Cardinium hertigii* | 16S rDNA | Primer | CLO-F: 5'-GGAACCTTACCTGGGCTAGAATGTATT-3'; CLO-R: 5'-GCCACTGTCTTCAAGCTCTACCAAC-3' | [56] |
|  |  | Protocol-1 | 95.0℃2min, then 94.0℃30s, 53.3℃30s 72.0℃2min in 30 cycles, 72.0℃ 4min |  |
|  |  | Protocol-2 | 95.0℃3min, then 95.0℃10s, 53.3℃30s, 72.0℃25s in 39 cycles, 72.0℃30s |  |
| *Fritschea bemisiae* | 16S rDNA | Primer | U23-F: 5'-GATGCCTTGGCATTGArAGGCGATGAAGGA-3'; 23sIG-R: 5'- TGGcTcATCATGcAAAAGGCA-3' | [35] |
|  |  | Protocol-1 | 95.0℃2min, then 94.0℃30s, 50.0℃60s, 72.0℃2min in 35 cycles, 72.0℃ 4min |  |
|  |  | Protocol-2 | 95.0℃3min, then 95.0℃10s, 48.3℃30s, 72.0℃25s in 39 cycles, 72.0℃30s |  |
| *Hamiltonella defensa* | 16S rDNA | Primer | 92F: 5'-TGAGTAAAGTCTGGGAATCTGG-3'; 1343R: 5'-CCCGGGAACGTATTCACCGTAG-3' | [33] |
|  |  | Protocol-1 | 95.0℃2min, then 94.0℃30s, 52.6℃45s, 72.0℃2min in 35 cycles, 72.0℃4min |  |
|  |  | Protocol-2 | 95.0℃3min, then 95.0℃10s, 58.5℃30s, 72.0℃25s in 39 cycles, 72.0℃30s |  |
| *Rickettsia* | 16S rDNA | Primer | RB-F: 5'-GCTCAGAACGAACGCTATC-3'; RB-R: 5'-GAAGGAAAGCATCTCTGC-3' | [57] |
|  |  | Protocol-1 | 95.0℃ 2min pre-denaturation, 94.0℃ 30s, 47.8℃ 30s, 72.0℃ 2min in 30 cycles, 72.0℃ 4min final extension |  |
|  |  | Protocol-2 | 95.0℃ 3min pre-denaturation, 95.0℃ 10s, 48.5℃ 30s read plate, 72.0℃25s in 39 cycles, 72.0℃30s melt curve |  |
| *Wolbachia* | 16S rDNA | Primer | 315F: 5'-GCATGAGTGAAGAAGGCC-3', 628R: 5'-AGATAGACGCCTTCGCCA3' | [58] |
|  |  | Protocol-1 | 95.0℃2min, then 94.0℃30s, 50.0℃45s, 72.0℃2min in 30 cycles, 72.0℃4min |  |
|  |  | Protocol-2 | 95.0℃3min, then 95.0℃10s, 55.0℃30s, 72.0℃25s in 39 cycles, 72.0℃30s |  |
