# Supplementary material for: Inactivation of Wolbachia Reveals Its Biological Roles in Whitefly Host
Source: PLoS One. 2012 Oct 29;7(10):e48148. doi: 10.1371/journal.pone.0048148 (PMC3483251; doi:10.1371/journal.pone.0048148)
Supplement: Method S1 — Fluorescence in situ hybridization of Bemisia tabaci samples. (DOC) [file pone.0048148.s006.doc]

**Method S1^#^**

***Fluorescence in situ hybridization of Bemisia tabaci samples.***

Whitelfy specimens were first fixed in Carnoy’s fixative (chloroform:ethanol: glacial acetic acid, 6:3:1) overnight at 4°C. The samples were then decolorized in 6% H_2_O_2_ in ethanol for 6 h and then hybridized overnight in hybridization buffer (20 mM Tris-HCl pH 8.0, 0.9 M NaCl, 0.01% sodiumdodecyl sulfate, 30% formamide) containing 10 pmol of fluorescent probes/ml at 37°C. Two 5’end *Wolbachia* probes W1: 5’-AATCCGGCCGARCCGACCC-3’ and W2: 5’-CTTCTGTGA GTACCGTCATTATC-3’ targeting the 16S rDNA were labeled with rhodamine. Stained samples were thoroughly washed twice in the same buffer at 48°C for 20 min, the adult whitefly, eggs and nymphs were mounted using the DAPI kit at 42°C for 30 min.
